# Transverse laparostomy is feasible and effective in the treatment of abdominal compartment syndrome in severe acute pancreatitis

**DOI:** 10.1186/1749-7922-3-6

**Published:** 2008-01-30

**Authors:** Ari Leppäniemi, Panu Mentula, Piia Hienonen, Esko Kemppainen

**Affiliations:** 1 Department of Gastroenterological and General Surgery, Meilahti hospital, University of Helsinki, Helsinki, Finland

## Abstract

**Background:**

Only recently has the important role of abdominal compartment syndrome (ACS) been recognized as a contributing factor to the multiple organ failure commonly seen in severe acute pancreatitis (SAP). Decompressive laparostomy for ACS is a life-saving procedure usually performed through a midline incision followed by a negative pressure wound dressing. High risk of intestinal fistulas and frequent inability to close the fascia with ensuing planned ventral hernia has prompted the search for alternative techniques. Subcutaneous fasciotomy may be effective in early and less severe cases of ACS but it is always accompanied with a ventral hernia.

**Case report:**

A patient with SAP developed manifest ACS and was treated with bilateral subcostal laparostomy. Immediately after decompression, the intra-abdominal pressure dropped from 23 mmHg to 10 mmHg, and the respiratory, cardiovascular and renal functions improved markedly leading to full recovery. The abdominal incision including the fascia and the skin was closed gradually over 4 relaparotomies, and during the 6 months' follow up there are no signs of ventral hernia or other wound complications.

**Discussion:**

Transverse subcostal laparostomy is a promising alternative decompression technique for ACS in SAP. It is feasible, effective and might provide a chance of early fascial closure. Comparative studies are needed to define its role as a decompressive technique for ACS.

## Background

Abdominal Compartment Syndrome (ACS) is defined as a state of serious organ dysfunction resulting from sustained increase in intra-abdominal pressure (IAP), that most obviously affects the cardiovascular, respiratory and renal systems [1]. Recently it has been suggested that some patients with severe acute pancreatitis (SAP) who develop early Multiple Organ Dysfunction Syndrome (MODS) in effect suffer from ACS caused by massive fluid resuscitation, capillary leak and visceral edema [2-7]. Although percutaneous drainage of ascites can in some cases decrease IAP at least temporarily, surgical decompression, most commonly performed through a long vertical midline incision leaving the abdomen open and covered with a negative abdominal pressure dressing, is the preferred method of treatment [8]. However, a literature analysis of 18 studies with 250 patients with ACS showed that although decompression has a significant effect in lowering IAP, mortality still remains considerable and little is known on its effect on organ dysfunction, highlighting the importance of careful consideration before surgical intervention [9]. In some cases, the poor response might be due to an intervention performed too late, but until more data is available, the decision for surgical decompression has to be individualized.

The open abdomen itself is associated with increased resource utilization and grave morbidity, especially increased risk of enteric fistulas. In a recent review of series of reports with at least 50 patients with open abdomen, the fistula rate among survivors varied from 0% to 50% being 3.6–6.7% in larger series consisting of trauma patients [10]. In addition, the risk of not being able to achieve fascial closure creating a hernia is considerable, 34–86% in mostly trauma patients [11-13], but the situation in patients with severe acute pancreatitis might be different, although poorly reported. The need to avoid open abdomen has lead to the search of alternative decompression techniques such as the subcutaneous linea alba fasciotomy [14]. Although effective in some patients, it automatically creates a ventral hernia that requires subsequent repair.

We present a patient with SAP where ACS was treated with transverse subcostal decompressive laparostomy and subsequent gradual delayed primary closure of the abdominal wall.

## Case Report

**Figure 1:**
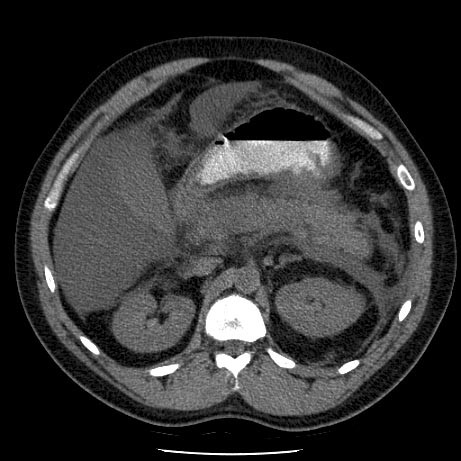
**CT scan on admission**. Computed tomography on admission shows peripancreatic oedema, thickening of the Gerota's fascia on the left side and at least two poorly defined fluid collections (in other cuts) corresponding to severe acute pancreatitis with Balthazar grade E [21].

A 29 years-old man with a medical history of traumatic cervical spine fracture (C6) managed operatively with full recovery and otherwise healthy presented to the emergency department with a one day history of epigastric pain and vomiting following excessive use of alcohol during the past 6 months. Based on physical examination (epigastric tenderness and guarding) and elevated plasma amylase levels (6 times normal upper limit), a diagnosis of alcohol-induced acute pancreatitis was made, the ultrasound examination revealed no gallstones or bile duct dilatation, and there was marked edema around the tail of the pancreas. The initial C-reactive protein level on admission was normal, but increased on the subsequent days to 205 mg/l (Day 1), and 271 mg/l (Day 2), respectively. The diagnosis and severity of acute pancreatitis were verified with a CT scan (Fig. 1). The initial treatment consisted of analgesics and intravenous crystalloid infusion and the patient was admitted to the emergency ward for observation.

The patient received 7000 ml of crystalloids during the first 12 hours and 7018 ml of crystalloids for the next 12 hours totaling more than 14 liters during the initial 24-hour period. The Ranson score was 4, and the APACHE II scores were 10 (admission day), 13 (Day 1) and 12 (Day 2), respectively. During the admission day, the blood pressure was 162–182/95–113, heart rate 122–130/min and the urine output dropped to 50 ml/hour. The pH was 7.31 and base excess (BE) -4.9. Because of increasing breathing difficulties requiring intubation and mechanical ventilation, anuria, need for norepinephrine (0.4 microgram/kg/min) and BE decreasing to -8, the patient was moved to a Surgical Intensive Care Unit (SICU) 16 hours post-admission.

IAP was measured via the urinary bladder initially 17 hours post-admission and was 27 mmHg. Subsequently, IAP was measured 8 times during the next 20 hours with values of 23–33 mmHg. The simultaneous mean arterial pressures (MAP) varied from 75 to 130 mmHg with calculated abdominal perfusion pressures (MAP-IAP) of 46–100 mmHg with a decreasing trend during the observation period.

**Figure 2:**
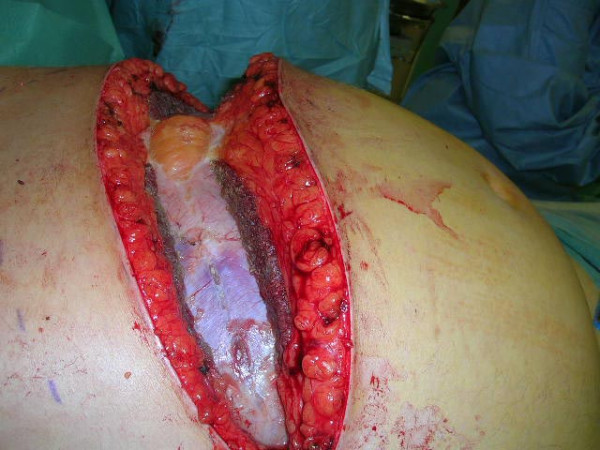
**Transverse laparotomy incision**. Seen from the patient's right side, a bilateral subcostal incision is being performed with posterior fascia still intact.

**Figure 3:**
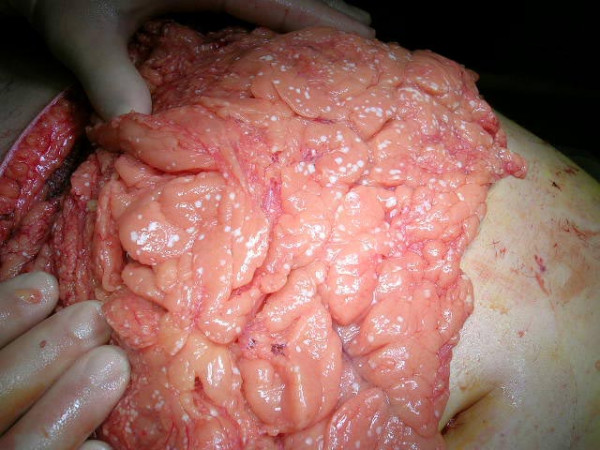
**Completed laparostomy**. Patches of liponecrosis seen on the surface of the greater omentum.

Because of increasing severity of organ dysfunction in spite of maximal supportive care and elevated IAP, a diagnosis of ACS was made and a decompressive laparostomy was performed 38 hours post-admission. A bilateral, wide subcostal incision about 5 cm below the costal margins was made and the fascial and muscle layers extending to about 5–8 cm lateral to the rectus sheats were divided on both sides (Fig. 2). Immediately before the incision, the IAP measured on the table was 23 mmHg dropping to 10 mmHg immediately after, before extensive drainage of ascites (about 2 liters). Simultaneously without changing the ventilator set up, the tidal volume increased from 400 to 500 ml, and the mean arterial pressure about 10 mmHg. The abdomen or peripancreatic area were not explored further (Fig. 3) and the viscera were covered with a negative pressure dressing. Back at the SICU a renal replacement therapy was started.

During the next 6 hours, the patient's condition improved markedly (norepinephrine down to 0.1 microgram/kg/min, BE -3, pulmonary function was improved and spontaneous diuresis started at 40–90 ml/hour). The abdominal dressing was changed 48 hours later and a tracheostomy was performed. Subsequent dressing changes were performed every 48–72 hours and every time about 3–5 cm of the fascia was closed from both edges with 1 polyglactin interrupted sutures. At the fourth dressing change, 10 days from decompression, the fascial and skin closures were complete without developing recurrent ACS, although the IAP rose from 15 to 24 mmHg, but causing no adverse effects requiring re-decompression. The patient was gradually getting better, was decannulated on day 16 post-admission and was transferred to a regular ward on day 20. The total length of SICU stay was 19 days. The patient made full recovery and was discharged 5 days later for a total hospital length of stay of 25 days. Seen at outpatient department 1 and 6 months later, the patient had made full recovery, there was no diabetes and the wound had healed without hernia or infection.

## Discussion

The mortality in SAP has decreased markedly in the last 20 years due to better understanding of the pathophysiology, early aggressive fluid resuscitation, timely surgical intervention, and aggressive monitoring and organ supporting intensive care [15]. However, in some patients the massive fluid resuscitation leads to visceral edema and ACS, with an estimated prevalence of 10%, although the prevalence of the milder form, intra-abdominal hypertension, is much higher, up to 78% in a retrospective study (IAP > 15 mmHg) and 25% in a prospective study (IAP > 25 cmH_2_O) [3-6].

The standard treatment after exhausting conservative management options is decompressive midline laparostomy. Although percutaneous drainage of pancreatic ascites, when present, can sometimes be helpful at least as a temporizing method, it was not considered sufficient in this patient and was not performed preoperatively.

Midline decompressive laparostomy is effective in decreasing IAP, rapid and easy to perform, but it is associated with a high risk of intestinal fistulas (about 5% in trauma patients with open abdomen) [10], and in many cases failure to close the fascia requiring complex reconstructive surgery 9–12 months later [16]. The subcutaneous approach eliminates the open abdomen, but might not be effective enough, as suggested by our own experience in 7 patients so far, where the decrease of IAP was sufficient in 4 patients. In addition, the subcutaneous fasciotomy always results in a ventral hernia requiring repair later on. Clinical experience also shows that early closure of the open abdomen is accompanied with improved clinical, nutritional and infection situation when the "catabolic drain" of the open wound is closed [17].

Transverse laparostomy is a promising alternative to the two techniques described above. A shown in the presented case, it was effective in treating the ACS, and gradual fascial closure could be performed with complete closure in 4 reoperations. Early follow up revealed no wound complications or hernia formation. It must be emphasized, however, that the overall incisional hernia rate in a large study of 2,983 laparotomy patients was 4.3% with 32% developing during the first 6 months [18]. Whether the transverse incision has a lesser incidence of incisional hernia formation is not known, and the follow up period in the presented case is obviously still short.

Although the transverse laparostomy takes slightly longer to perform than midline laparostomy, same principles of open abdomen management can be applied without additional equipment. The major disadvantage could be the loss of abdominal and back extensor muscle functions, if fascial closure could not be achieved. This might require complex reconstruction procedures including innervated free flaps that not only restore continuity but also the functional integrity of the abdomen [19].

In SAP, infected pancreatic necrosis is an established indication for surgical necrosectomy, ideally postponed until four weeks after the onset of symptoms and performed most commonly through a transverse midline incision [20]. Although the need for necrosectomy has decreased markedly in the last years, the transverse incision used for decompressive laparostomy could also be used for necrosectomy avoiding multiple incisions and associated surgical and cosmetic disadvantages. Whether standard midline laparostomy at least in patients with SAP should be replaced with transverse laparostomy requires further comparative studies.

## Competing interests

The author(s) declare that they have no competing interests.

## Authors' contributions

AL: Acquisition, analysis and interpretation of the data, drafting the manuscript; PM: Interpretation of the data, revising the manuscript critically; PH: Acquisition of the data, revising the manuscript critically; EK: Revising the manuscript critically. All authors have read and approved the final manuscript. No funding involved.
